# Supplementary material for: Weight change-related factors during the COVID-19 pandemic: a population-based cross-sectional study using social cognitive theory
Source: PeerJ. 2022 Jul 27;10:e13829. doi: 10.7717/peerj.13829 (PMC9338751; doi:10.7717/peerj.13829)

Appendix 1: A diagram representing the survey variables mapped to the three pillars of the Social Cognitive Theory

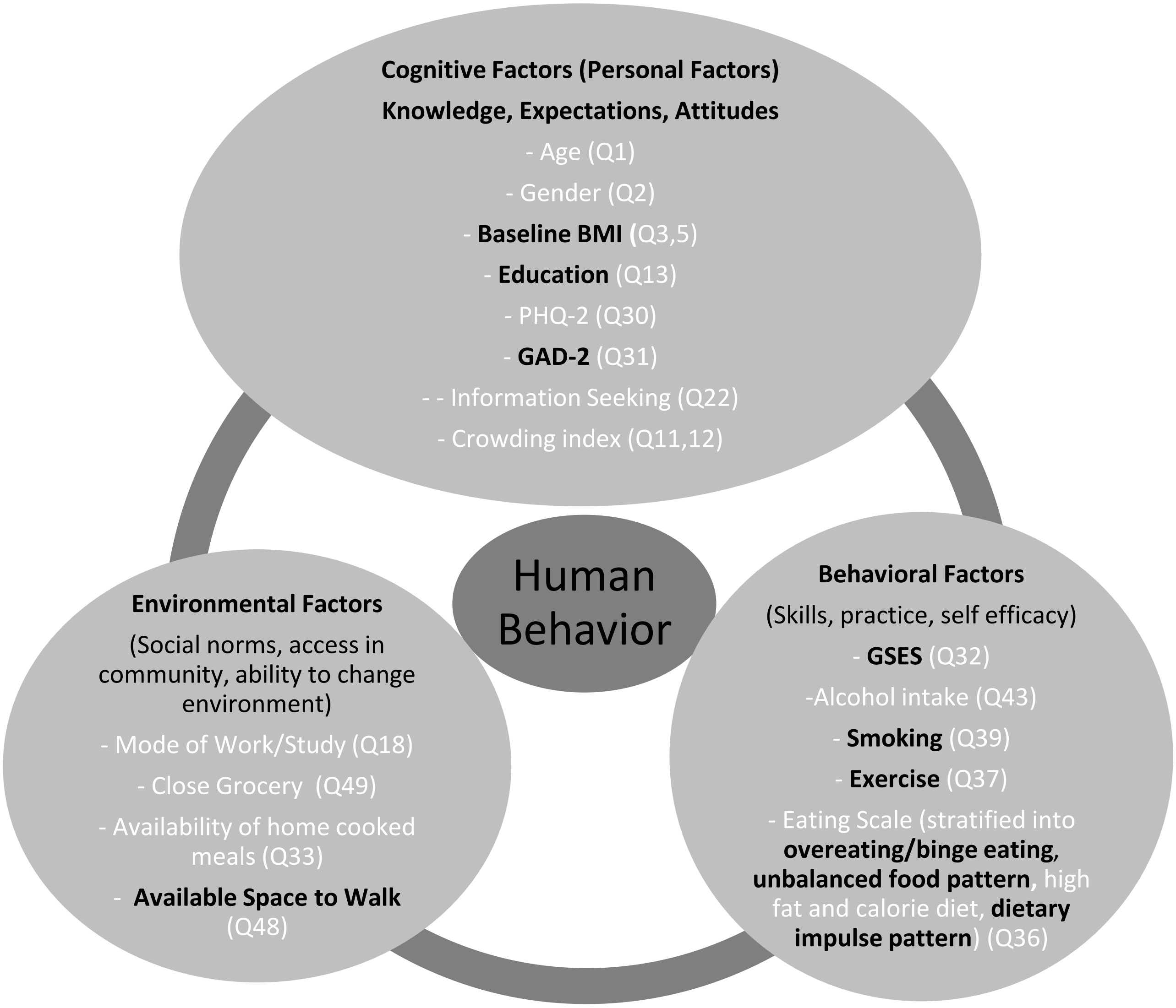

Supplement: Supplemental Information 2 [file peerj-10-13829-s002.pdf]
