# Supplementary material for: Weight change-related factors during the COVID-19 pandemic: a population-based cross-sectional study using social cognitive theory
Source: PeerJ. 2022 Jul 27;10:e13829. doi: 10.7717/peerj.13829 (PMC9338751; doi:10.7717/peerj.13829)
Supplement: Supplemental Information 3 [file peerj-10-13829-s003.pdf]

Questionnaire

Demographics

1- **Age:** _____ **years**

2- **Gender:**

1. Female
2. Male

3- **Weight before the COVID-19 pandemic (March 2020) (in Kg):** --------

4- **Current weight (in Kg):** -------------

5- **Height (in cm):** --------------

6- **How did your weight change during the COVID-19 pandemic?**

1. Stayed the same
2. Increased
3. Decreased
4. Fluctuated

7- **Marital Status**

1. Single
2. Married
3. In a relationship
4. Widowed
5. Divorced

8- **How many children do you have?** (Please type zero if none or not applicable)

Please specify: ______

9- **In which governorate (Muhafaza) do you live?**

1. Akkar
2. Baalbek-Hermel
3. Beirut
4. Beqaa
5. Mount Lebanon

Institutional Review Board
American University of Beirut

15 JUN 2021

APPROVED

6. Nabatieh
7. North
8. South

10- <u>Where do you live?</u>

1. Major city
2. Village
3. Suburbs

11- <u>How many individuals live in the same house as yours?</u> (excluding newborn infants)

Please specify: ______

12- <u>How many rooms are there in your house</u>? (excluding kitchens and bathrooms)

Please specify: __________

13- <u>Education</u>

1. No education
2. Below high school
3. High school or technical school
4. University degree/Post graduate

14- <u>How did your work status change during the COVID-19 pandemic?</u>

1. Did not change
2. I was unemployed and got a job
3. I became unemployed
4. I changed my job

15- <u>What's your current work status?</u>

1. Unemployed (if so, please skip questions 16 and 17)
2. Self employed
3. Employed
4. Student (if so, please skip question 16)
5. Both employed and a student

Institutional Review Board
American University of Beirut

15 JUN 2021

APPROVED

16- **<u>How much did you earn per month in the past 1 year? (in LBP)</u>**

1. Zero
2. < 1,000,000
3. 1,000,000 – 2,999,999
4. 3,000,000 – 4,999,999
5. 5,000,000 – 7,000,000
6. > 7, 000, 000

17- **<u>Since the start of the COVID-19 pandemic, how were you working/studying?</u>**

1. Working or studying from home
2. Commuting to work or university
3. A combination of both

18- **<u>During the multiple TOTAL LOCKDOWN periods imposed by the government, how were you working/studying?</u>**

1. Working or studying from home
2. Commuting to work or university
3. A combination of both

19- **<u>Were you infected by the COVID-19 virus since March 2020?</u>**

1. Yes
2. No, if so, please skip question 20

20- **<u>Did you require hospitalization?</u>**

1. Yes
2. No

21- **<u>Do you have a chronic medical condition for which you take medications on daily basis?</u>**

1. Yes
2. No

<u>Cognitive factors (Knowledge, Expectation, Attitude)</u>

22- **<u>What is your *main* source of information on COVID-19?</u>**

<u>Please select the most appropriate answer</u>

Institutional Review Board
American University of Beirut

15 JUN 2021

APPROVED

1. Social media (Facebook, Instagram, Twitter, etc..)
2. Browsing the internet
3. TV/Radio
4. Newspaper (including online newspapers)
5. Colleagues (if your colleague is a health care professional, please choose answer #6)
6. Health care professionals (Doctor, nurse, pharmacist)
7. Friends/ Family members

23- <u>How often did you search for information linking COVID-19 to overweight or obesity?</u>

1. Always
2. Frequently
3. Sometimes
4. Seldom
5. Never

24- <u>How often did you search for information on how to control weight during the COVID-19 pandemic and multiple lockdowns imposed by the government?</u>

1. Always
2. Frequently
3. Sometimes
4. Seldom
5. Never

25- <u>How far do you agree or disagree with the following?</u>

	**Strongly Agree**	**Agree**	**Neutral**	**Disagree**	**Strongly Disagree**
I am concerned about my weight because it may affect my health.					
I am concerned about my weight because it affects my physical appearance.					

Institutional Review Board
American University of Beirut

15 JUN 2021

APPROVED

Weight gain makes individuals prone to disease (heart disease, diabetes, hypertension, etc..).					

26- Were you diagnosed with any of the following *before* the COVID-19 pandemic?

1. Anorexia Nervosa
2. Bulimia Nervosa
3. Binge Eating Disorder
4. Generalized Anxiety Disorder
5. Major Depressive Disorder
6. Other: please specify:
7. None

27- Were you diagnosed with any of the following *during* the COVID-19 pandemic?

1. Anorexia Nervosa
2. Bulimia Nervosa
3. Binge eating disorder
4. Generalized Anxiety Disorder
5. Major Depressive Disorder
6. Other: please specify:
7. None

28- Throughout the COVID-19 pandemic, I had concerns related to the economy, including fear of being laid off or experiencing poorer personal economy.

29- Throughout the COVID-19 pandemic, I experienced fear or anxiety related to the COVID-19 virus.

1. Always
2. Very Frequently
3. Occasionally
4. Rarely
5. Very Rarely
6. Never

Institutional Review Board
American University of Beirut

15 JUN 2021

APPROVED

30- **<u>Over the past two weeks, how often have you been bothered by any of the following?</u>**

	Not at all	Several days	More than half the days	Nearly every day
**Little interest or pleasure in doing things.**	0	1	2	3
**Feeling down, depressed, or hopeless.**	0	1	2	3

<u>Warning:</u> Please add your scores on the above two questions and if you score 3 or more please visit your primary care physician for formal assessment or kindly contact the AUBMC psychiatry department on 01-350000 extension: 5650.

31- **<u>Over the past two weeks, how often have you been bothered by the following?</u>**

	Not at all	Several days	More than half the days	Nearly every day
**Feeling nervous, anxious or on edge**	0	1	2	3
**Not being able to stop or control worrying.**	0	1	2	3

<u>Warning:</u> Please add your scores on the above two questions and if you score 3 or more please visit your primary care physician for formal assessment or kindly contact the AUBMC psychiatry department on 01-350000 extension: 5650.

32- **<u>General Self-Efficacy Tool</u>**

Institutional Review Board
American University of Beirut

15 JUN 2021

APPROVED

<u>For each of the following items below, please rate your answer with:</u>
<u>1: not at all true, 2: hardly true, 3: moderately true or 4: exactly true</u>

	**Not at all true**	**Hardly true**	**Moderately true**	**Exactly true**
I can always manage to solve difficult problems if I try hard enough.	**1**	**2**	**3**	**4**
If someone opposes me, I can find the means and ways to get what I want.	**1**	**2**	**3**	**4**
It is easy for me to stick to my aims and accomplish my goals.	**1**	**2**	**3**	**4**
I am confident that I could deal efficiently with unexpected events.	**1**	**2**	**3**	**4**
Thanks to my resourcefulness, I know how to handle unforeseen situations.	**1**	**2**	**3**	**4**
I can solve most problems if I invest the necessary effort.	**1**	**2**	**3**	**4**
I can remain calm when facing difficulties because I can rely on my coping abilities.	**1**	**2**	**3**	**4**
When I am confronted with a problem, I can usually find several solutions.	**1**	**2**	**3**	**4**
If I am in trouble, I can usually think of a solution.	**1**	**2**	**3**	**4**
I can usually handle whatever comes my way.	**1**	**2**	**3**	**4**

<u>Behavioral Factors</u>

33- **<u>How often are home cooked meals available to you?</u>** (including home cooked meals prepared by yourself, others or ordered from restaurants)

1. Always

Institutional Review Board
American University of Beirut

15 JUN 2021

APPROVED

2. Very Frequently
3. Occasionally
4. Rarely
5. Very Rarely
6. Never

34- **<u>How did you have access to food during the periods of LOCKDOWN imposed by the government</u>?** Please select all that apply.

1. Online shopping/Delivery services
2. Nearby shops
3. Municipality distributed food

35- **<u>How did the frequency of *consuming/ordering fast food* change during the COVID-19 pandemic compared to before?</u>**

1. Increased
2. Decreased
3. Stayed the same
4. I do not order fast food at all

<u>3</u>

	**Not at All**	**No**	**Average**	**Yes**	**Very much so**
Do you continue to eat even after you feel like stopping?	**1**	**2**	**3**	**4**	**5**
Do you overeat frequently?	**1**	**2**	**3**	**4**	**5**
Do you eat more food than you had planned to?	**1**	**2**	**3**	**4**	**5**
Do you feel depressed or regret after overeating?	**1**	**2**	**3**	**4**	**5**
Do you feel that you do not have a good dietary behavior as compared to other people?	**1**	**2**	**3**	**4**	**5**
Do you overeat when you eat out? (except cafeteria staff)	**1**	**2**	**3**	**4**	**5**
Do you sometimes eat food although you are not hungry?	**1**	**2**	**3**	**4**	**5**
Have you ever failed in trying to lose weight?	**1**	**2**	**3**	**4**	**5**
Can you stop having a meal when you are full?	**1**	**2**	**3**	**4**	**5**
Do you often eat instant noodles?	**1**	**2**	**3**	**4**	**5**
Do you often eat meat products such as ham or sausage?	**1**	**2**	**3**	**4**	**5**

Institutional Review Board
American University of Beirut

15 JUN 2021

APPROVED

Do you eat bread, pizza and chicken instead of your regular meal at dinner?	**1**	**2**	**3**	**4**	**5**
Do you eat fast food more than twice a week?	**1**	**2**	**3**	**4**	**5**
Do you usually eat out (except restaurant staff) about twice a week?	**1**	**2**	**3**	**4**	**5**
Do you like greasy food?	**1**	**2**	**3**	**4**	**5**
Do you often eat greasy food such as fried food, stir-fry, or salad dressing?	**1**	**2**	**3**	**4**	**5**
How often do you eat snacks instead of a regular meal?	**1**	**2**	**3**	**4**	**5**
Do you eat fried or stir-fry dishes at least once in 2 days?	**1**	**2**	**3**	**4**	**5**
Do you eat foods containing sugar (cracker, candy, chocolates, cakes, ice cream) every day?	**1**	**2**	**3**	**4**	**5**
Do you drink soft drinks, vitamin drinks, or fruit juice instead of water when you feel thirsty?	**1**	**2**	**3**	**4**	**5**
Do you like meat?	**1**	**2**	**3**	**4**	**5**
Do you eat vegetables in each meal?	**1**	**2**	**3**	**4**	**5**
Are you eating beans (rice with bean, bean paste stew, or stewed bean in soy cause) or tofu once a day?	**1**	**2**	**3**	**4**	**5**
Are you having fruits or fruit juice every day?	**1**	**2**	**3**	**4**	**5**
Are you eating at least one of the following foods: meat, fish, egg, bean, and tofu when you have a meals?	**1**	**2**	**3**	**4**	**5**
Do you prefer to eat multi-grain rice over white rice?	**1**	**2**	**3**	**4**	**5**
Do you eat food when you feel depressed or unhappy?	**1**	**2**	**3**	**4**	**5**
Do you eat food when you feel inferior or dissatisfied?	**1**	**2**	**3**	**4**	**5**
Do you eat food on impulse?	**1**	**2**	**3**	**4**	**5**
Do you eat food when you feel anxiety or stress?	**1**	**2**	**3**	**4**	**5**
Do you feel that you should eat delicious food when you get stressed?	**1**	**2**	**3**	**4**	**5**
Do you eat food when you are angry?	**1**	**2**	**3**	**4**	**5**
Do you feel that you can recover yourself through eating sugary foods?	**1**	**2**	**3**	**4**	**5**
Do you eat food when you feel bored?	**1**	**2**	**3**	**4**	**5**

Institutional Review Board
American University of Beirut

15 JUN 2021

APPROVED

36- **<u>During the COVID-19 pandemic, did you visit or follow up with a nutritionist or physician in regard to your weight management?</u> (whether in person or online)**

1. Yes
2. No

37- **<u>How did your exercise habits change during the COVID-19 pandemic?</u>**

1. I increased my daily physical activity
2. I decreased my daily physical activity
3. Stayed the same
4. I used to exercise and became sedentary
5. I already live and continue to live a sedentary lifestyle

38- **<u>On average, how many hours of sleep did you get per night during the COVID-19 pandemic?</u>**

1. < 4 hours
2. 4-5 hours
3. 6-7 hours
4. 8 hours and more

39- **<u>Do you smoke?</u>**

1. Yes
2. No (if so, skip questions 40 and 41)

40- **<u>What is the approximate number of cigarettes (per pack) that you smoke per day?</u>**

Please specify: _____________ pack/day

41- **<u>What is the number of hours you spend on smoking hubble bubble per week?</u>**

Please specify: ____________ hours

Institutional Review Board
American University of Beirut

15 JUN 2021

APPROVED

43- Do you drink alcohol?

1. Yes
2. No (if so, please skip question 44)

44- Please answer the following questions about alcohol intake and calculate your score:

	**0**	**1**	**2**	**3**	**4**	**Score**
**How often do you have a drink containing alcohol?**	never	monthly or less	2 to 4 times a month	2 to 3 times a week	4 or more times a week	
**How often do you have more than five or more drinks on one occasion?**	never	monthly or less	2 to 4 times a month	2 to 3 times a week	4 or more times a week	
**How many drinks containing alcohol do you have on a typical day when you are drinking?**	1 or 2	3 or 4	5 or 6	7 to 9	10 or more	
					**Total Score**	

Warning: If you a score 3 (for females) or 4 (for males) please consult your primary care physician for formal assessment as this may be an indication of hazardous drinking or alcohol use disorder, or kindly contact the AUBMC psychiatry department on 01-350000 extension: 5650.

Institutional Review Board
American University of Beirut

15 JUN 2021

APPROVED

Environmental Factors

45- During the COVID-19 pandemic, how often did you visit your neighbors or family members?

1. Never
2. Seldom
3. Sometimes
4. Frequently
5. Always

46- During the COVID-19 pandemic, how often did you attend social gatherings?
(including meeting with 2 or more people that are not the people you live with such as visiting neighbors and/or friends, conferences/meetings that are not online, festivities, or weddings)

1. Never
2. Seldom
3. Sometimes
4. Frequently
5. Always

47- During the COVID pandemic LOCKDOWNS, did you have a space in your neighborhood where you can walk outdoors?

1. Yes
2. No

48- Do you have a nearby accessible grocery store?

1. Yes
2. No

Institutional Review Board
American University of Beirut

15 JUN 2021

APPROVED

Thank you so much for taking the time in order to fill this questionnaire.

For tips on losing weight offered by the National Health Service of the United Kingdom, kindly press on the following link that will redirect you.

https://www.nhs.uk/live-well/healthy-weight/12-tips-to-help-you-lose-weight/

Institutional Review Board
American University of Beirut

15 JUN 2021

APPROVED
